# Personalized immunization to optimize vaccine immunogenicity: exploring the multidimensional effects of host intrinsic factors, external intervention strategies, and the external environment

**DOI:** 10.3389/fimmu.2025.1655819

**Published:** 2025-08-27

**Authors:** Keda Chen, Siyi Gu, Jiaxuan Li, Yutong Xu, Zhiyi Wang, Yanjun Zhang, Jianhua Li

**Affiliations:** ^1^ Key Laboratory of Artificial Organs, Computational Medicine in Zhejiang Province, Shulan International Medical College, Zhejiang Shuren University, Hangzhou, China; ^2^ Zhejiang Key Laboratory of Public Health Detection, Pathogenesis Research, Department of Microbiology, Zhejiang Provincial Center for Disease Control and Prevention, Hangzhou, China

**Keywords:** vaccine, immunogenicity, metabolism, genetics, acupuncture, moxibustion, near-infrared light therapy

## Abstract

Vaccines are a fundamental tool in the prevention and control of infectious diseases; however, significant individual variability in immunogenicity exists. This variability is not solely attributable to vaccine characteristics but is also influenced by a range of factors. This review systematically examines the key factors influencing vaccine immunogenicity, with particular emphasis on host-intrinsic factors (e.g., metabolic status, genetic background), personalized external interventions, such as optimized vaccine delivery techniques (e.g., aspiration-based skin delivery) and immunomodulatory adjuvant therapies (e.g., acupuncture, moxibustion, and near-infrared light therapy), as well as environmental exposures and immune memory. By examining the mechanisms and recent research advancements associated with these factors, this paper seeks to provide a foundation for the development of personalized vaccination strategies to address future public health challenges.

## 1 Introduction

Immunogenicity refers to the ability of a vaccine to activate the body’s immune system and induce a targeted immune response. In simple terms, it denotes the extent to which a vaccine stimulates the immune system to produce antibodies and/or initiate cell-mediated immune responses. This property is a crucial indicator for evaluating vaccine efficacy, as it is directly linked to the vaccine’s capacity to provide protection against the target pathogen. Immunogenicity can be assessed by indicators such as antibody positivity (defined as the presence of antibodies at week 8 post-vaccination in pre-immunization negative populations) and the geometric mean titer (GMT) of HAV-IgG (1).

Infectious diseases, such as SARS, Ebola, and influenza, are marked by rapid transmission and high contagiousness, representing an ongoing threat to global public health (2). Vaccines, which serve as the primary tool for prevention and control, play an indispensable role in reducing both morbidity and mortality. However, their effectiveness can be influenced by individual variations, limiting their overall impact.

**Figure 1:**
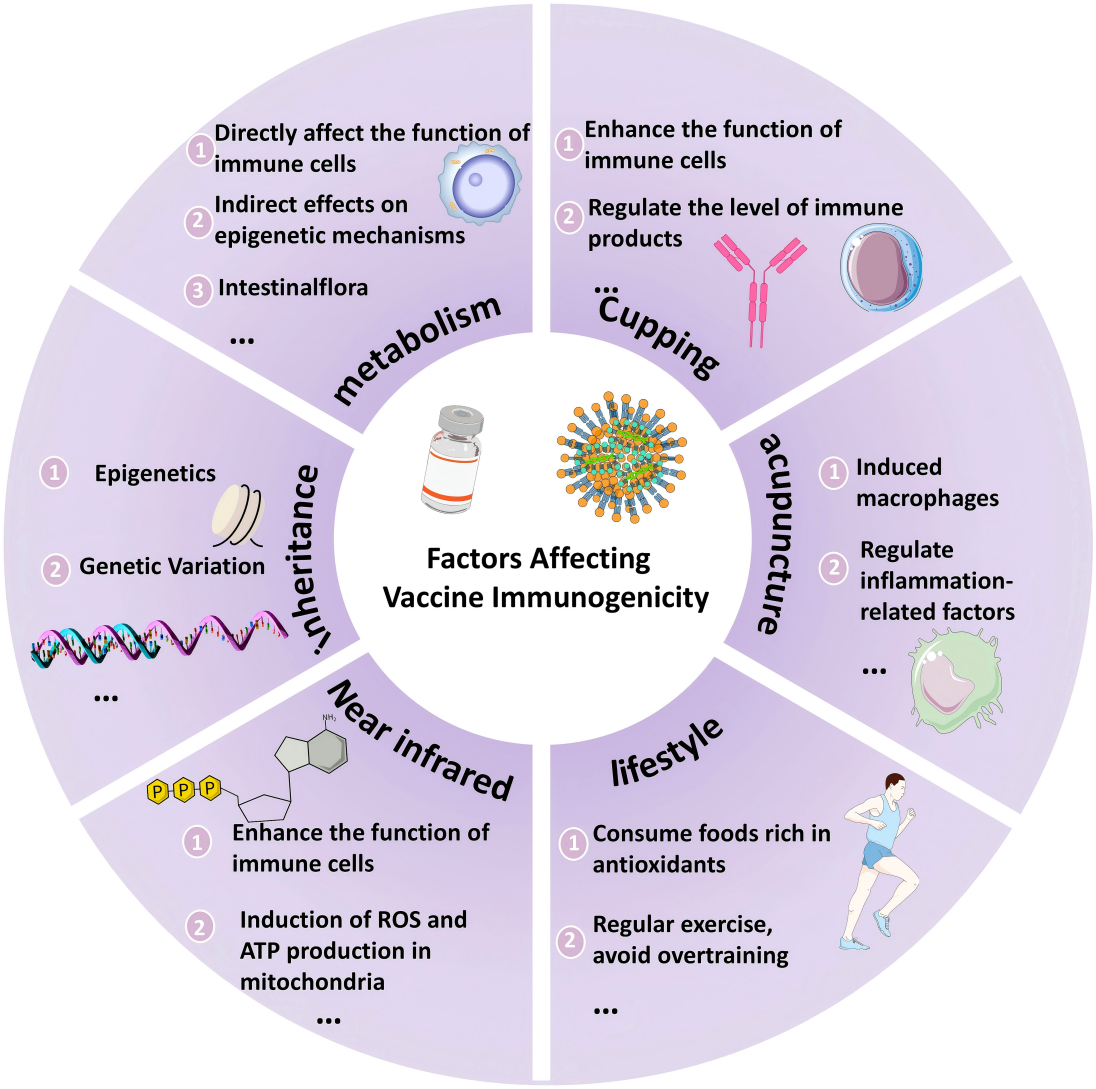
Multidimensional influences on vaccine immunogenicity. This schematic systematically summarises the key factors influencing vaccine immunogenicity, including the following core dimensions: 1) Individual characteristics. Metabolic state: gut flora regulates immune cell function through metabolites that support T cell memory formation; metabolic pathways (e.g., glycolysis, mitochondrial function) directly influence CD8^+^ T cell activity. 2) Genetic background. Genetic variants (e.g. VDR, GC gene polymorphisms) and epigenetic regulation (DNA methylation, m6A modifications) lead to inter-individual differences in immune response. 3) Novel drug delivery technologies. Aspiration-based skin delivery: the negative pressure environment enhances the depth of skin penetration of the DNA vaccine, promotes antigenic expression, increases antibody titers (with a significant increase in GMT values) and avoids the risk of tissue damage from electroporation.4) complementary therapy. Acupuncture and moxibustion: activation of the autonomic-endocrine-immune axis, down-regulation of pro-inflammatory factors (IL-6, TNF-α), up-regulation of anti-inflammatory factors (IL-10, TGF-β), restoration of the Th1/Th2 balance, and enhancement of HSP70 expression for antigen presentation.5) Near-infrared light therapy. Through the absorption of light energy by mitochondrial cytochrome c oxidase, it induces the production of ROS and ATP, activates the release of chemokines (CCL2, CCL20) from mast cells, promotes the migration of dendritic cells to lymph nodes, and enhances the effect of vaccine adjuvant.6) Nutrition and Lifestyle. High-fat diets and circadian rhythm disruption suppress immune function, whereas antioxidant diets (vitamin C, zinc) and moderate exercise improve immune homeostasis.

This heterogeneity is not only influenced by antigen design, but is also strongly associated with host-intrinsic factors (genetic background, metabolic status, etc.), external intervention strategies (delivery technologies, adjuvant therapies, dietary and lifestyle interventions), and environmental exposures. Consequently, this review aims to systematically analyze the mechanisms by which multidimensional factors influence immunogenicity and to provide a foundation for the development of personalized vaccine strategies. (see **Figure 1**).

## 2 Influence of host intrinsic factors on the immune response to vaccines

### 2.1 Changes in immune function regulated by metabolic state

Cellular metabolic processes serve dual physiological roles, extending beyond their primary function of supplying essential energy to encompass the modulation of immunological mechanisms. Between populations, the metabolic status of an individual can significantly influence the functioning of his or her immune system due to differences in genetic background, environmental factors, and lifestyle. These differences become especially pronounced in the context of viral infections such as COVID-19 (3).

During SARS-CoV-2 infection, the metabolic profile of patients is altered, as indicated by a reduction in oxygen partial pressure and oxygen saturation, which directly affects the metabolic activity of peripheral immune cells (4, 5). In individuals with acute SARS-CoV-2 infection, CD8+ T lymphocytes isolated from peripheral blood exhibit significant metabolic disturbances, including reduced glycolytic activity, diminished activity of the mTOR signaling pathway and compromised mitochondrial efficiency (6). The impairment of T-cell functionality resulting from these metabolic irregularities could consequently contribute to an escalation in the pathological manifestations of the condition. In addition, some specific T-cell subsets exhibit high protein abundance levels of the voltage-gated anion-selective channel 1 (VDAC1) located within mitochondria and H3K27me3 chromatin markers, features that correlate with disease severity and are more susceptible to mitochondrial apoptosis, which may lead to lymphopenia (7). Thus, accumulating evidence suggests that impaired T-cell functionality is significantly correlated with clinical progression in COVID-19 patients. This pathophysiological connection implies that therapeutic interventions targeting lymphocyte regulation could potentially enhance clinical outcomes for individuals experiencing acute COVID-19.

Besides directly influencing immune cell function, metabolic status also indirectly regulates immune responses via epigenetic mechanisms. For instance, the co-regulatory role of α-KG in enhancing the enzymatic activity of JMJD3, a histone demethylase, is pivotal for modulating the differentiation processes of T lymphocytes. Alpha-KG from glutamine metabolism is able to alter the chromatin structure of effector functionally relevant gene loci, thereby affecting mTOR signaling (8). Furthermore, DNA methyltransferase DNMT3A and the demethylase TET2 are involved in the epigenetic reprogramming of T cells (9). These enzymes collaborate in T-cell memory formation, despite having opposing roles in DNA methylation. These findings underscore how metabolites can influence the fate of immune cells via epigenetic modifications.

### 2.2 Genetic diversity and individual differences in immune response

The impact of genetic factors on an individual’s immune response is both intricate and multifactorial, affecting not only the individual’s ability to fight infections, but also determining the effectiveness of vaccinations and susceptibility to autoimmune diseases. T lymphocytes as key mediators of adaptive immunity, are essential for coordinating targeted immune reactions., and their function is profoundly influenced by epigenetic regulation. This regulation is achieved through epigenetic regulatory processes such as histone modification and DNA methylation, which are tightly associated with metabolic pathways.

Firstly, T cells undergo a series of epigenetic and metabolic changes as the aging process progresses across different age groups. Among aging individuals, T lymphocytes show alterations in the global genomic methylation profiles, in particular the CpG islands of repressed genes are highly methylated, while the surrounding regions show a hypomethylated state (10). This implies that specific gene expression may be tightly suppressed or activated during senescence, thereby affecting the functional activity of T cells. Consequently, the immune system of older individuals typically exhibits diminished function and a reduced capacity to respond to emerging pathogens.

In recent years, several new advances have been made in epigenetic regulation in influencing the individual immune system. Deletion of the N6-methyladenine (m6A) demethylase FTO promotes CD8+ T memory precursor cell (TMP) differentiation and elevates anti-tumor immune response 2.1-fold through enhancement of the STAT5 signaling pathway (11). Methyltransferase (METTL3) plays a crucial role in m6A modification, regulating immune-related signaling pathways (12, 13). METTL3-mediated m6A modification preserves Th17 cellular epigenetic memory by stabilizing Tcf7 mRNA (Δ half-life +3.5 hours) and is strongly associated with relapse in autoimmune diseases (14).

Additionally, genetic diversity accounts for a substantial portion of the variation in immune response among individuals. Genetic variations that exist among different populations may cause certain individuals to be more susceptible to developing chronic inflammatory diseases or to exhibit stronger or weaker immunity against viral or bacterial infections. Vitamin D, for instance, plays an essential role in the proper functioning of the immune system (15). Specifically, genetic alterations in two critical loci - the vitamin D receptor (VDR) gene and the group-specific component (GC) gene encoding vitamin D-binding protein (VDBP) - have been identified as potential contributors to increase susceptibility to asthma (16). LIN28, an evolutionarily conserved protein with RNA-binding activity, serves as a key modulator of multiple post-transcriptional regulatory mechanisms in cellular systems. Breast cancer susceptibility appears to be associated with genetic variation in the Lin28B gene, and haplotypes in this region are associated with increased risk (17).

These findings underscore the pivotal role of epigenetic factors and genetic variation in immunity. Understanding these mechanisms can help develop more effective personalized vaccine strategies, especially for the elderly and genetically susceptible populations. By adjusting epigenetic regulation and optimizing metabolic pathways, vaccine-induced immune responses can be enhanced and the overall efficacy of vaccines improved. This not only offers novel insights into vaccine design, but also establishes a foundational framework for precision medicine and personalized vaccination.

### 2.3 Intestinal flora and its metabolites and immune regulation

Intestinal microbiota and their metabolites play a crucial role in immunomodulation through various mechanisms. Short-chain fatty acids (SCFAs), including acetic acid, propionic acid, and butyric acid, function as key metabolites by inhibiting histone deacetylase (HDAC) and promoting the expression of Foxp3, a critical gene for regulatory T cell (Treg) differentiation (18). Additionally, SCFAs activate G-protein-coupled receptors (GPR43/41), suppressing the inflammatory pathways of Th17 cells (18), and enhance the oxidative phosphorylation of CD8+ T cells to drive the transformation of memory precursor cells to tissue-resident memory T cells. cells to tissue-resident memory T cells, extending the vaccine protection period by 40% (19). Dysbiosis, characterized by reduced levels of Muribaculaceae and the absence of Clostridium butyricum, results in a decline in SCFAs, which disrupts the intestinal barrier and promotes systemic inflammation, such as Th17/Treg imbalance observed in preeclampsia (20, 21). Restoration of SCFAs levels by fecal transplants (FMT) or probiotics significantly improves the immune response (22). Therefore, targeting flora metabolites is a potential strategy to enhance vaccine efficacy.

### 2.4 Effect of age, sex and other key variables on immune response

In addition to metabolic status and genetic background, a variety of intrinsic host factors significantly influence vaccine immunogenicity, including age, gender, nutritional status, and underlying disease state. Together, these factors shape an individual’s ability to mount an immune response.

Several studies have shown that vaccine immunogenicity generally decreases with age. For example, the BNT162b2 mRNA vaccine produced significantly lower levels of antibodies in the elderly than in the younger population (23). However, booster doses can partially restore immunogenicity in older populations to levels comparable to those in younger groups (24). The immune response to vaccines in infants and young children is often suboptimal and may be related to immature immune system development (25). For example, measles-rubella (MR) vaccines differ in immunogenicity between 6- and 9-month-old infants (1). Age and body mass index (BMI) may jointly influence the immune response, such as in influenza vaccines, where age and BMI interact on hemagglutination inhibition (HAI) antibody levels (26).

Several studies have noted that women are generally more immunogenic to vaccines (e.g., mRNA vaccine, shingles vaccine) than men (27). For example, HIV vaccine trials have shown stronger immune responses in female-born individuals (AFAB) (28). Gender differences may be related to sex hormones (e.g., estrogen and testosterone) as well as genetic factors (e.g., HLA genotype) (29). For example, women are more likely to develop anti-drug antibodies when using infliximab (30).

The effect of nutritional status on vaccine immunogenicity is a complex process with multifactorial effects. In hemodialysis patients, malnutrition inflammation score (MIS) was negatively correlated with antibody response to BNT162b2 vaccine (Pfizer mRNA vaccine), suggesting that moderate-to-severe malnutrition reduces humoral immune response (31). Low-protein diets diminish the protective effect of malaria vaccines (e.g., radiation attenuated spore vaccine RAS), leading to impaired immune cell differentiation and tissue-resident immune response (32). Deficiencies in micronutrients (e.g., vitamins C, D, zinc, selenium) may impair vaccine responses. For example, iron deficiency (affecting 2 billion people globally) is associated with reduced vaccine response, especially in infants, pregnant women and the elderly (33, 34).

Vaccine immunogenicity is lower in diabetic patients (especially those with poor glycemic control), whereas antibody responses are stronger in patients with better renal function (GFR) (35). The immune response to vaccines (e.g., CoronaVac) is weaker in patients with autoimmune rheumatic diseases (ARD) and is affected by immunosuppressive agents (e.g., infliximab) (36, 37). Vaccine immunogenicity may be reduced in patients with chronic obstructive pulmonary disease (COPD) (38), HIV infection (at CD4 counts <500/μL) (39), and cancer (40).

## 3 External intervention strategies to optimize vaccine immunogenicity

**Figure 2:**
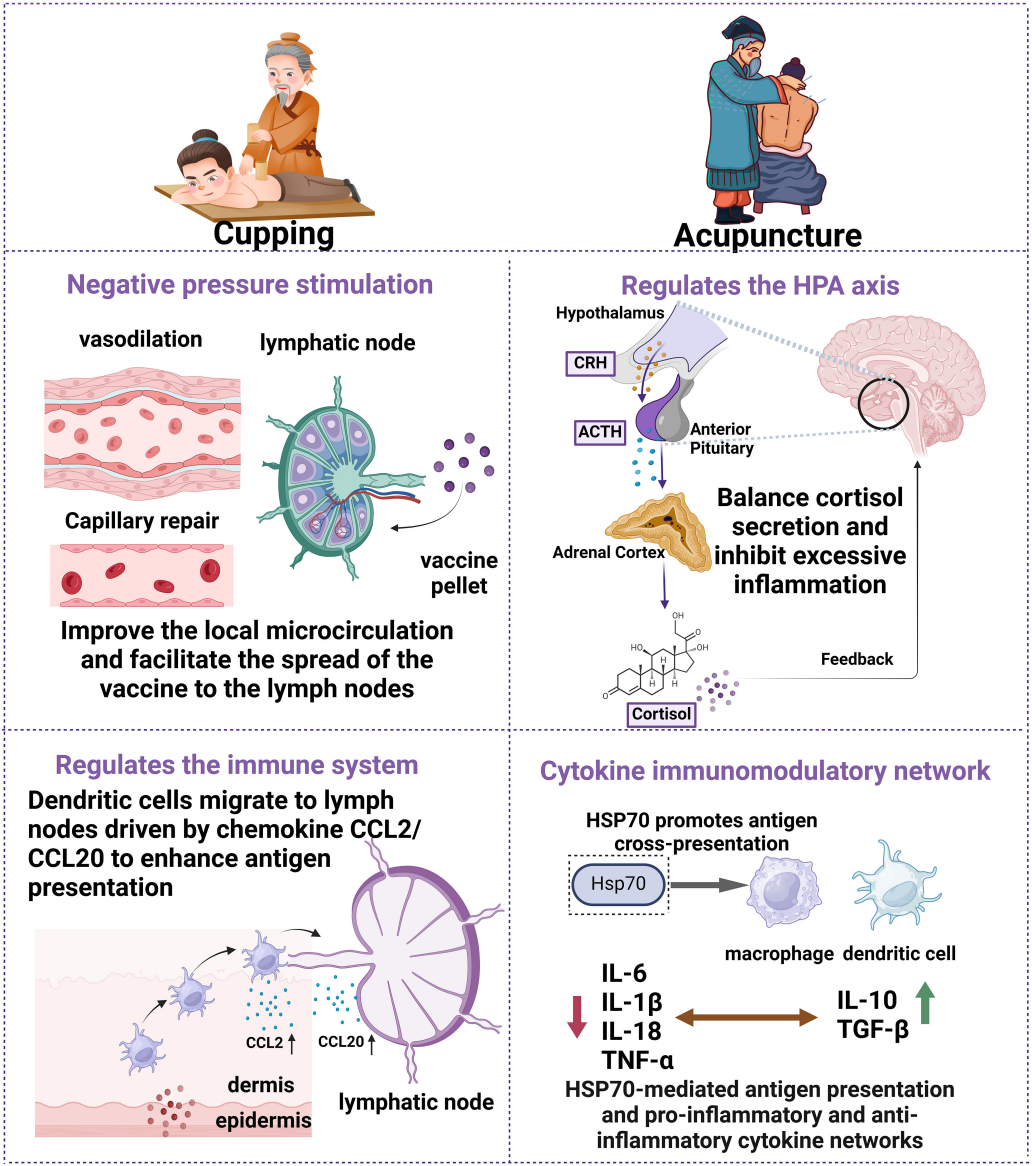
Schematic diagram of the mechanism of cupping and acupuncture in regulating immune response. Cupping (left side) negative pressure stimulation causes vasodilatation, repairs capillary endothelium, improves local microcirculation, and promotes rapid diffusion of vaccine particles to lymphatic vessels and lymph nodes. Up-regulation of chemokine CCL2 and CCL20 expression drives dendritic cell (DC) migration to local lymph nodes and enhances antigen presentation efficiency. Acupuncture (right side) activates the hypothalamic-pituitary-adrenal axis (HPA axis) by stimulating specific points (e.g., ST36, CV4, GV20). Balancing cortisol secretion and suppressing excessive inflammation via a CRH-ACTH feedback loop. Inhibits pro-inflammatory factors (IL-6, IL-1β, TNF-α) and increases levels of anti-inflammatory factors (IL-10, TGF-β). Enhanced expression of heat shock protein 70 (HSP70) mediates antigen cross-presentation through the CD91/LOX-1 receptor.

Consideration of individual metabolic and genetic differences is essential to optimize vaccine efficacy when exploring personalized immune boosting strategies. While traditional vaccination methods have achieved significant results in preventing infectious diseases, their effectiveness varies significantly among different populations. To overcome this challenge, researchers are exploring a variety of innovative ways to enhance the immune response to vaccines. This section will focus on several emerging strategies for personalized immune enhancement, including optimization of vaccine delivery techniques, immune-assisted therapies and dietary and lifestyle interventions. These approaches not only improve the immunogenicity of vaccines, but may also lead to more precise and efficient vaccination results by modulating the metabolic state and immune function of individuals (see **Figure 2**).

### 3.1 Optimizing vaccine delivery technology: aspiration-based skin delivery

#### 3.1.1 Physiological mechanisms and immune activation

Cupping induces localized inflammation on the surface of the skin by applying subatmospheric pressure, thereby stimulating the immune system and enhancing blood circulation. This therapy dilates blood vessels, promotes local blood flow, improves microcirculation, repairs capillary endothelial cells, and accelerates tissue repair and angiogenesis. Improved blood circulation helps to eliminate toxic substances and metabolic byproducts from the organism, promoting muscle relaxation and the normalization of overall functional status (41, 42). Additionally, cupping may enhance the release of endogenous opioids, improve pain management, and promote a sense of overall comfort and relaxation (43).

Activation of the immune system by cupping include enhancement of the function of immune cells and modulation of the level of immune products. Existing research indicates that cupping therapy exerts modulatory effects on immune-related biomarkers. Specifically, this intervention has been observed to elevate neutrophil counts in peripheral circulation while simultaneously downregulating the concentrations of serum immunoglobulin E (Ig E) and interleukin-2 (IL-2). Furthermore, there is a notable upregulation of complement component C3 levels in the bloodstream. These coordinated immunological changes highlight cupping’s capacity to regulate cellular immune functions and optimize humoral immune responses, thereby contributing to the enhancement of host defense mechanisms through multiple biological pathways (44). Cupping also upregulates immunomodulatory factors such as interferon (IFN-γ) and tumor necrosis factor (TNF), improving the immune system’s defenses. Moreover, the stimulation of the thymus gland by cupping promotes the flow of lymphatic fluid and enhances the circulation and function of immune cells. Cupping may also further enhance the activity of the immune system by regulating immunoglobulin levels, improving the immunological roles of red blood cells, and promoting the production of histamine-like substances (45, 46). After stimulating the skin surface, the micro-environmental changes triggered by cupping will also be converted into biological signals through the neuroendocrine system, further activating the immune system and producing therapeutic effects (47).

Overall, cupping produces a multifaceted physiological effect on the body through a variety of pathways, including improving blood circulation, regulating the immune system, and influencing the autonomic nervous system and endocrine system. These mechanisms not only help to improve immune function, but may also have positive preventive and therapeutic effects on chronic diseases such as diabetes and cardiovascular disease. Although studies have been done to support these effects of cupping, further scientific research is needed to fully understand its specific mechanisms and clinical applications.

#### 3.1.2 Impact on the effectiveness of vaccination

Emran O’Lallow et al. developed a transdermal delivery platform utilizing vacuum technology for delivering genetic material into living organisms that mimics traditional Chinese cupping therapy and Middle Eastern hijama practices (48, 49). Conventionally, the direct administration of uncomplicated plasmid DNA exhibits suboptimal transfection efficacy, necessitating supplementary delivery enhancement strategies including lipid-based cationic vectors, polymeric carriers, electrophoretic membrane destabilization techniques, biolistic particle-mediated approaches, and hydrodynamic microinjection systems to augment both transgene expression levels and subsequent immunological responses. Among these adjuvanted methodologies, electrophoretic permeabilization(EP) technology has emerged as the predominant strategy adopted in contemporary biomedical research due to its technical reproducibility and dose-dependent response characteristics (50–53). Statistical analysis of recent clinical research data reveals that EP demonstrates prevalent utilization in nucleic acid-based therapeutic applications (54). Although electrical stimulation via EP systems shows therapeutic potential, certain physiological responses and clinical limitations must be considered. At the administration sites, this intervention may induce localized adverse effects including nociceptive reactions, involuntary muscular contractions, and potential cellular trauma. Furthermore, certain patient populations require special precautions. Individuals with implanted electronic medical devices (e.g., cardiac pacemakers or automated defibrillators) may experience hazardous interactions from the applied electrical currents (55–58), in addition to the high cost of supplementary equipment. The suction-based skin delivery technique, which does not require current stimulation and is better tolerated compared to electroporation, is particularly suitable for people who are sensitive to electroporation (59), which can optimize the delivery efficiency of different vaccines (e.g., H1N1, SARS-CoV-2 nanovaccines) or individual skin characteristics by modulating the intensity of the negative pressure and the duration of action (59). Simultaneous synergy with microneedle (e.g., MN@EV/SC system) or iontophoresis technology can further enhance the depth of penetration and dose control of large molecules (e.g., DNA, mRNA vaccines) (60, 61).

Emran O’Lallow’s team experimentally investigated the effects of cupping (i.e., the use of a suction device to create a negative-pressure environment) in vaccine delivery, with a particular focus on its impact on delivery efficiency and immune response (62). According to the statistical analysis conducted in this investigation, no significant differences were observed in delivery effect by suction time in the range of 5 to 300 seconds. However, aspiration immediately after injection promotes the expression of the target antigen (GFP-labelled) more rapidly than injection only, with punctate expression detected as early as 1 hour at the skin margins and extending over time to the epidermis and upper dermis. After 24 hours, GFP expression in the aspiration group reached its maximum intensity and was mainly concentrated at a subsurface depth measuring around 400 microns relative to the epidermal surface, much deeper than in the injection alone group (63). For serum ELISA, 96-well plates were coated with SARS-CoV-2 S1-Fc protein (1.5 μg/mL), incubated with serum gradient dilution, and HRP-labeled secondary antibody (rabbit anti-rat IgG, 1:2000) was used for color development (62). Endpoint potency was defined as OD_492_ > baseline mean + 2.5 SD inverse of the highest dilution (62). SARS-CoV-2 antibody levels were determined by ELISA, and the results showed that the GMT (titer) of the humoral response after the use of the aspiration device ranged from 2×10^3 to 5×10^3, the experimental measurements demonstrated markedly elevated values when compared to those observed in the sole administration group. In addition, rats receiving a single injection plus aspiration produced an immune response similar to that of rats receiving two injections. HE staining (hematoxylin-eosin staining) analysis did not reveal any local tissue damage or lymphocyte infiltration, suggesting good biocompatibility of the method. The phenomenon of localized tissue damage or lymphocyte infiltration is not only common in EP, but is considered necessary to ensure an immune response (64, 65).

Given the complex interplay between innate immune function and metabolic balance, empirical studies suggest that cupping therapy may influence immunological responses through secondary pathways, potentially mitigating disease-related physiological disturbances (66). A randomized clinical trial by Ahmed et al. (2005) demonstrated that the application of bloodletting cupping (BLC) as a complementary therapeutic intervention in the management of rheumatoid arthritis (RA).Specifically, the therapeutic modality showed statistically significant reductions in serum C-reactive protein (CRP) concentrations and demonstrated observable improvement in erythrocyte sedimentation rate (ESR) measurements compared to control groups. Early decreases in these laboratory values were superior to conventional treatments (67). In a healthy population, wet cupping resulted in decreased NK cell activity and cytotoxicity and increased NK cell levels in RA patients (67). It was found that in healthy people, after wet cupping, the expression of activation receptors (e.g., CD54, CD69) of NK cells was down-regulated, thus inhibiting their cytotoxic function (67, 68); wet cupping may indirectly inhibit the activity of NK cells by removing inflammatory mediators in the blood or by regulating the secretion of cytokines from immune cells (e.g., monocytes) (69, 70). For example, the reduced levels of heavy metals (e.g., aluminum, zinc, cadmium) in the blood after wet cupping may attenuate the stimulation of NK cells by oxidative stress (71). In RA patients, wet cupping reduces the sustained activation and depletion of NK cells by removing body fluids containing high inflammatory factors (e.g., TNF-α, IL-15) (72, 73); wet cupping may promote the expansion of CD56bright NK cells (which have a stronger immunomodulatory function), thereby inhibiting the role of pro-inflammatory CD56dim NK cells (74), while it may downregulate inhibitory PD-1 and other inhibitory receptors, reversing the depleted state of NK cells (75, 76). This suggests that cupping modulates both innate immune responses (NK cells) and acquired cell-mediated immunological reactions (e.g. sIL-2R).

Notably, a randomized controlled clinical trial (NCT05901337) of COVID-19 convalescent patients further explored the modulatory effects of cupping therapy on human immune parameters. The study included 76 recovered patients with T-lymphocytopenia, cytokine dysregulation and low immunoglobulin (IgA/IgM/IgG), who were randomly divided into two groups: an intervention group (traditional medication + a single session of dry cupping therapy) versus a control group (traditional medication only). T-cell subsets, serum cytokines and immunoglobulin levels were analyzed by flow cytometry. Although the final results are not yet available, its rigorous design (MANOVA-based sample size calculation, α=0.05, effect size 0.39) directly validates the potential of cupping to modulate the human immune system and provides a clinical translational rationale for suction-based skin delivery techniques.

This innovative methodology utilizing aspiration technology establishes a cutaneous administration system for biomolecular agents and prophylactic formulations. The developed platform demonstrates significant advantages in operational simplicity, economic feasibility, and production scalability while ensuring both biosafety requirements and therapeutic efficacy standards are maintained, which generates high levels of transgene expression and enhances vaccine immunity.

### 3.2 Immunomodulatory adjuvant therapy

#### 3.2.1 Acupuncture and moxibustion

##### 3.2.1.1 Physiological mechanisms and immune activation

The regulatory mechanisms of acupuncture on immune function primarily involve its neuroregulatory effects, which are mediated through stimulation of the hypothalamic-pituitary-adrenal (HPA) axis and activation of autonomic nerve pathways. Specifically, acupuncture enhances the secretion of certain neuromodulators and increases the expression of glucocorticoids and their receptors, thereby modulating immune function (77). Furthermore, acupuncture exhibits immunomodulatory effects by regulating the pathophysiological microenvironment in inflammatory lesions. This therapeutic intervention downregulates the expression of key proinflammatory mediators including IL-6, IL-1β, IL-18, and TNF-α, while simultaneously upregulating the bioactivity of anti-inflammatory regulators exemplified by IL-10 and TGF-β (78).

Existing clinical investigations have demonstrated that the application of acupoint stimulation therapy exhibits notable efficacy in managing various inflammatory pathologies. Specifically, this therapeutic modality has been shown to improve rheumatic joint disorders, nasal hypersensitivity reactions, and gastrointestinal inflammatory conditions in both acute and chronic stages. Mechanistic studies suggest its anti-inflammatory properties primarily stem from the downregulation of key pro-inflammatory signaling molecules, such as IL-6 and TNF-α, thereby modulating immune-mediated pathological processes. The observed cytokine profile alterations correlate with significant attenuation of clinical manifestations in multiple trial cohorts. Additionally, acupuncture has been demonstrated to slow disease progression and improve symptoms in animal models of autoimmune diseases, including multiple sclerosis (79, 80).

The mechanisms underlying the activation of immune responses through acupuncture involve promoting the phenotypic transition of macrophages from proinflammatory M1 variants to immunoregulatory M2 subtypes, while simultaneously reducing the levels of pro-inflammatory factors such as TNF-α and IL-6 (81).It also increases the concentration of anti-inflammatory factors such as IL-10 and TGF-β. In addition, scientific investigations have demonstrated that acupuncture exerts immunomodulatory effects by suppressing the activity of pro-inflammatory lymphocyte subsets, particularly Th1 and Th17 populations, along with their corresponding interleukin secretions. Furthermore, this therapeutic intervention enhances the activity of immunosuppressive Tregs and stimulates the production of anti-inflammatory mediators including TGF-β. These coordinated cellular responses effectively restore the equilibrium between Th1 and Th2 cell populations within the immune system (79, 80). These effects contribute to maintaining the immune system’s steady state against pathogen invasion and abnormal immune responses.

##### 3.2.1.2 Impact on vaccination efficacy

Jia Li et al. explored the effects of acupuncture treatment on d-galactose-induced vaccine-induced immune responses in senescent rats. The protective efficacy of vaccination is primarily correlated with the strength of immunological responses elicited in recipients. Notably, immunodeficient populations exhibit heightened susceptibility to suboptimal immunization outcomes, particularly among elderly individuals experiencing age-associated declines in adaptive immune functions (82–84).This vulnerable demographic frequently demonstrates compromised responsiveness in both lymphocyte-mediated defenses and antibody production mechanisms, resulting in diminished capacity to establish sufficient immunological memory against pathogenic challenges (85). Immunosenescence, a key pathological feature in aging, refers to the deterioration of the immune system with age, manifested by a progressive weakening of the adaptive immune response to external antigenic stimuli. The clinical manifestations of this pathological state are multidimensional: not only does it significantly weaken the body’s defense against exogenous pathogens, but it is also positively correlated with the risk of malignant tumors; at the same time, it may induce abnormalities in the immune tolerance mechanism, leading to an increase in the incidence of autoimmune diseases. It is worth noting that the imbalance of the immune microenvironment homeostasis also leads to a reduction in the efficacy of the immune response to vaccines, which is reflected in the molecular biology of decreased antigen presentation efficiency and insufficient production of protective antibodies (86). To overcome immune senescence, current research efforts are focused on the design and synthesis of novel immunological enhancers for vaccines (87) The team initially proposed acupuncture as an adjuvant for vaccine response. Acupuncture is considered to be one of the basic methods of preventing infectious diseases. Moreover, the mechanism by which acupuncture enhances the action of antigen-presenting cells and lymphocytes (88) is similar to that of adjuvants. Experimental data showed that moxibustion intervention significantly modulated the body’s immune response to Staphylococcus aureus (S. aureus) antigens in the vaccine, as well as promoting the increase of specific antibody potency in experimental animals. Notably, this immunomodulatory effect showed a dose-dependent feature in the rabbit model, and the mechanism may be closely related to the activation of intrinsic immune cells and the synergistic effect of humoral immune response (89).

The team allocated 40 female Wistar rats (230-280g, 10 weeks old) into four groups of 10 rats each. Group A simulated immunity plus acupuncture, Group B simulated immunity plus control, Group C normal immunity plus acupuncture, Group D normal immunity plus control. Rats in groups A and B were injected subcutaneously with d-galactose (350 mg/kg day), and groups C and D were injected with saline for 6 weeks. The model induced by d-galactose has been extensively employed in scientific investigations into aging mechanisms and related pathological conditions in rodents (90). Excessive accumulation of d-galactose induces oxidative stress through ROS generation while simultaneously impairing the activity of endogenous antioxidant defense systems within cerebral tissues. This dual mechanism subsequently triggers a cascade of neurophysiological alterations, manifesting as cognitive impairment, accelerated brain aging phenotypes, progressive declines in motor coordination, and diminished lifespan expectancy in experimental murine models. Notably, this pathophysiological progression effectively recapitulates key aspects of physiological aging observed in mammalian organisms (91). In this study, experimental rodent models were established through chronic administration of D-Galactose to induce physiological alterations. At the seventh week of the experimental timeline, all subjects received intramuscular administration of acellular pertussis-containing vaccine (DTaP) at a dosage equivalent to one-twentieth of the standard human immunization quantity. The DTaP immunization protocol implemented in this investigation has demonstrated clinical efficacy in both adolescent and adult populations (92); however, antibody titers in vaccine recipients diminish at a significant rate (93), In addition, the administration of DTaP vaccine has been linked to various adverse effects impacting the digestive system, such as nausea, vomiting, diarrhea and stomach pain. The research group hypothesizes acupuncture may improve the body’s immune response to vaccines by functioning in an adjuvant-like manner. This hypothesis is grounded in traditional Chinese medical principles regarding meridian regulation and empirical evidence from prior experimental investigations. Specifically, the therapeutic protocol involving simultaneous stimulation of Zusanli (ST36, Leg Three Li) (94), Guanyuan (CV4, Cinnabar Field), and Baihui (GV20, Hundred Convergences) acupoints - termed “dual tonification and single regulation” therapy - demonstrates particular potential, has a special effect on regulating the immune system and delaying immune aging in rats (95–97). Rats in groups A and C underwent electroacupuncture and moxibustion at specific acupoints (ST36, CV4, GV20), and the control group was fixed only.

For the assessment of their immune response, diphtheria antibody titers were detected using the Vero cell neutralization test (98). The experimental data revealed significant variations in diphtheria antitoxin levels among the different intervention groups. Quantitative analysis revealed that the immunization model control cohort exhibited markedly reduced antitoxin concentrations relative to the standard immunization control group, with statistical significance confirmed. Contrastingly, subjects receiving combined low-frequency electroacupuncture stimulation and thermal moxibustion therapy in the model intervention group displayed substantially enhanced antitoxin production, achieving statistical significance. Furthermore, the conventional acupuncture intervention group demonstrated a measurable improvement in the immunological response, although the effect size was relatively modest.

Previous studies have demonstrated that the liquid formulations of the DTaP vaccine exhibit cytotoxic effects on cells. Consequently, researchers employed quantitative analysis of lymphocyte subpopulations to indirectly assess the effects mediated by cellular immunity. Experimental observations revealed enhanced apoptotic tendencies in T-lymphocytes within aging organisms, with CD4+ lymphocytes demonstrating higher apoptotic susceptibility compared to CD8+ counterparts. This selective depletion resulted in marked numerical reduction of CD4+ populations, consequently inverting the typical CD4+/CD8+ ratio within the immune cell repertoire (99). Quantitative analysis of lymphocyte subpopulations was conducted using flow cytometry to assess the distribution patterns of CD3+, CD4+, and CD8+ cells. Comparative analysis revealed a marked disparity in CD4+/CD8+ T lymphocyte ratios among CD3+ populations across experimental cohorts. The model immunization control cohort exhibited a statistically significant reduction in this immunoregulatory index relative to the standard immunization control group (p<0.01). Subsequent therapeutic application of electroacupuncture combined with moxibustion modalities in the model intervention group demonstrated a notable elevation in this critical immune parameter, reaching statistical significance at p<0.05 when compared with pretreatment baseline measurements. The CD4T/CD8T cell ratio was significantly lower in group B model immunized controls compared to group D normal immunized controls. The group A model immunized with acupuncture had a higher CD4T/CD8T cell ratio compared to the group B model immunized control. The CD4T/CD8T cell ratio was increased in normal-immunized acupuncture compared with normal-immunized controls (p < 0.05). The assessment of diphtheria antitoxin titers and CD4+ to CD8+ T lymphocyte ratios currently serves as validated biomarkers for evaluating immune responsiveness in humans following DTaP immunization. Nevertheless, the therapeutic potential of combined electroacupuncture and moxibustion to modulate these immunoregulatory parameters remains insufficiently explored in clinical research (100, 101).

Heat shock proteins (HSPs), serving as essential molecular chaperones, are crucial for maintaining proteostasis and structural integrity within cellular systems. These conserved polypeptides demonstrate significant immunomodulatory capacities through their involvement in coordinating diverse immune cell populations, while concurrently facilitating antigen processing and presentation mechanisms essential for adaptive immunity (102, 103). The secretion of HSPs is triggered by cellular stress and exposure to immune danger signals. Once released into the extracellular compartments or systemic circulation, HSPs interact with cellular membranes via receptor-mediated mechanisms, triggering intracellular signaling pathways that promote the internalization of immunogenic polypeptide. This biological process initiates a cascade of molecular events, ultimately promoting the efficient processing and presentation of antigenic determinants within the host immune system (104). HSP70-bound peptides can be effectively internalized by antigen-presenting cells (105) via CD91, LOX-1 (106, 107), thereby facilitating the presentation of the antigenic peptide via major histocompatibility complex (MHC) class I molecules on the cell surface. The team determined the expression level of HSP70 mRNA in spleen tissues by real-time RT-PCR, and used GAPDH as an internal reference to analysis gene expression. The analysis of splenocyte gene expression profiles revealed a marked reduction in HSP70 transcriptional activity among subjects subjected to model immunization protocols when contrasted with counterparts receiving standard immunization regimens (p < 0.01). However, the relative expression of HSP70 mRNA in splenocytes was elevated in the immunoacupuncture group after the model immunoacupuncture treatment (P < 0.05). Additionally, the relative expression of HSP70 mRNA in splenocytes showed a slight increase in the normal-immunized acupuncture C group compared to the normal-immunized control group D (P > 0.05).

The results showed significant increases in diphtheria antitoxin titer, CD4T/CD8T cell ratio and HSP70 mRNA expression after acupuncture intervention, suggesting that these practices could be used as novel vaccine adjuvants. Importantly, the DTaP vaccination protocol employed in this study utilized aluminum hydroxide as an immunostimulant, facilitating the concurrent evaluation of acupuncture’s potential adjuvant compatibility. These findings suggest that acupuncture may synergize with aluminum-based adjuvants in therapeutic applications, based on the experimental parameters employed. Nevertheless, constrained by the experimental parameters, this research presents two principal methodological limitations: first, the investigation did not include quantitative analysis of HSP70 protein expression levels through standardized immunoassay techniques; second, the temporal dynamics of immune activation processes remained unexplored across critical immunological phases.

Ulcerative colitis (UC), a persistent inflammatory disorder of the colonic mucosa, manifests as a chronic condition characterized by intermittent phases of mucosal inflammation and tissue damage. UC patients usually exhibit disruption of the Treg/Th17 immune axis. The therapeutic mechanism of moxibustion in traditional Chinese medicine involves suppression of SIRT1 protein activity, thereby modulating the immune homeostasis mediated by Th17/Treg cell differentiation. Experimental evidence indicates that concurrent administration of EX-527, a selective SIRT1 enzymatic antagonist, synergistically enhances the clinical efficacy of moxibustion intervention through coordinated immunoregulatory pathways (108). In addition, a study showed that acupuncture combined with bloodletting effectively inhibited mast cell degranulation and improved histopathological morphology in sensitized skin tissues on the back of rats with urticaria. The mechanism of action may be related to the inhibition of the differentiation and proliferation of helper T cells 2, as well as the regulation of humoral immune response (109).

Notably, a randomized controlled clinical trial (NCT04844710) of mild-to-moderate COVID-19 hospitalized patients has explored the immunomodulatory effects of acupuncture in combination with standard of care. The study directly assessed acupuncture’s ability to modulate the human immune system by testing laboratory markers (e.g., lymphocyte subsets, NK cell activity, and inflammatory factor levels). Although the final results are not yet available, its framework of research focusing on immune parameters provides an important rationale for the clinical translation of acupuncture to enhance vaccine response, especially in a population of recovering infections with poor vaccine response.

#### 3.2.2 Near-infrared phototherapy

##### 3.2.2.1 Physiological mechanisms and immune activation

Near-infrared (NIR) light therapy, also known as low-level laser therapy or photobiomodulation (PBM), is a non-invasive treatment that utilizes light at specific wavelengths to promote cellular healing and tissue restoration. NIR light radiates in a non-ionizing manner and does not cause significant tissue damage or genotoxicity (110). Equipment for NIR is much cheaper compared to radiation equipment (111). NIR lasers have been developed and utilized in the medical field for decades, with their safety and theoretical foundations well-established (112). Cytochrome c oxidase (CCO) in mitochondria is one of the main photoreceptors when NIR is irradiated to biological tissues. CCO absorbs photons and its activity is enhanced, optimizing the function of the electron transport chain, increasing ATP production and improving cellular energy metabolism. This process not only enhance cellular respiratory efficiency but also mitigates the formation of free radicals and alleviates oxidative stress by upregulating antioxidant enzymes, such as superoxide dismutase (SOD).

NIR is able to down-regulate the expression of inflammatory cytokines including TNF-α, IL-1β, and IL-6, while up-regulating the levels of anti-inflammatory mediators like IL-10, thereby reducing the inflammatory response and maintaining an appropriate immune response. This modulatory effect makes NIR excellent in treating many types of pain, such as arthritis, musculoskeletal pain, and post-operative pain, by reducing inflammation and promoting repair of damaged tissue to achieve pain relief. For chronic wounds such as diabetic foot ulcers and burns, NIR can accelerate the healing process and reduce the risk of infection.

Mechanisms of activation of the immune system by NIR include enhancement of immune cell function. By improving cellular energy metabolism, NIR contributes to maintaining the physiological activities of immune-related cellular components, including macrophages and lymphocytes, by regulating their functional integrity. In addition, NIR promotes macrophage polarization towards the M2 type, which exhibits enhanced anti-inflammatory and reparative properties. For T cells, NIR may enhance the proliferation and differentiation of CD4+ T cells, support Th1/Th2 balance, and stimulate the production of regulatory T cells (Treg), which play a critical role in suppressing autoimmune responses and maintaining immune tolerance. NIR also affects the immune system indirectly through neuroendocrine pathways, for example, it regulates the hypothalamic-pituitary-adrenal axis (HPA axis), which in turn modulates the secretion of immunomodulatory hormones like glucocorticoids.

##### 3.2.2.2 Effectiveness as a vaccine adjuvant

Although the concept of using lasers as vaccine adjuvants emerged in the 1960s, recent studies have identified mast cells as key mediators of their immunostimulatory effects; however, the precise cellular mechanisms, and in particular how light triggers mast cell activation, remain incompletely elucidated (113). Physiological and biological activities of 1270 nm near-infrared light as a vaccine adjuvant in a mouse model of influenza studied by Yohei Maki et al. The near-infrared photoadjuvant effect is wavelength specific. 1270 nm band is preferred based on the following molecular absorption properties (1): Oxygen molecule absorption matching: Under the partial pressure of oxygen in biological tissues, this band covers both the discrete absorption line of O_2_ monomer (1268 nm) and the continuous absorption band of O_2_-O_2_/O_2_-N_2_ collision complex (1264 nm) (114). Measured absorption cross sections showed that the peak absorption cross section of 21% O_2_ mixture at 294 K body temperature environment amounted to 2.52 × 10^-²⁶ cm²/molecule (monomer) versus 4.87 × 10^-¹⁹ cm^5^/molecule² (complex) (114). (2) Tissue penetration optimization: Near 1450 nm (one of the main absorption peaks of water), water molecules in tissues strongly absorb photons, resulting in a significant increase in light attenuation and a significant shortening of the effective attenuation depth (penetration depth) (115). In contrast, the linear absorption coefficient of water at 1270 nm (0.122 cm-¹) is much lower than that at 1450 nm (3.05 cm-¹), so the penetration depth can theoretically be increased by 25 times (116). (3) Mitochondrial energy matching: 1270 nm laser irradiation can directly generate single-linear oxygen (without photosensitizer), and its characteristic phosphorescence peak is located at 1270 nm, which can be detected by near-infrared spectroscopy (117); in neurons and astrocytes, 1270 nm laser-induced single-linear oxygen can elevate the mitochondrial membrane potential (ΔΨm), activate NADH/FADH_2_-dependent respiration, and increase the maximal respiration rate, which ultimately promotes ATP production (117); 1270 nm light is highly selective for the generation of single-linear oxygen species and does not induce the accumulation of harmful ROS such as superoxide anion or hydrogen peroxide (118).

Using the P-815 mouse mast cell tumor cell line, mastocytes play a critical role in modulating immunological processes (119)., ROS were detected with H2DCFDA (2’,7’-dichlorodihydrofluorescein diacetate), and the intensity of cellular fluorescence was analyzed following laser irradiation. Continuous-wave 1270 nm near-infrared laser irradiation at 300 m W/cm² markedly elevated DCF-positive cell populations associated with reactive oxygen species in *in vitro* cultured mast cells. Quantitative analysis revealed that cellular fluorescence parameters, particularly the median fluorescence intensity (MFI), exhibited a radiation intensity-dependent enhancement pattern when contrasted with untreated experimental controls. This systematic investigation provides evidence that continuous-wave near-infrared illumination within the 1270 nm spectrum stimulates intracellular reactive oxygen species generation in mast cell populations under specified irradiation conditions.

In eukaryotic organisms, ROS generation primarily occurs within the mitochondrial as a natural consequence of oxidative phosphorylation during aerobic respiration. Notably, there exists a significant correlation between the mitochondrial ATP synthesis machinery and the concomitant synthesis of reactive oxygen species within cellular systems (120). The concentration of ATP in both intact P-815 cellular models and purified mitochondrial fractions was determined using an enzymatic bioluminescence assay with luciferin-luciferase detection systems. Experimental data revealed that photonic stimulation at 1270 nm wavelength in complete cellular environments significantly augmented bioenergetic molecule biosynthesis, with observed metabolic enhancement demonstrating a direct proportionality to incident radiant flux density. From mitochondria isolated individually, ATP is produced in large quantities at both 1270 nm 200 m W/cm2 and 300 m W/cm2 wavelengths as it is in the whole cell. Experimental findings demonstrate that the mechanism by which laser adjuvants interact with mast cells under continuous-wave 1270 nm near-infrared light exposure originates from the mitochondrial generation of ATP and ROS. These organelle-derived biomolecules act as photosensitive signaling mediators initiating cellular responses to photonic stimulation. Six hours after laser irradiation of mouse skin, RNA was extracted and analyzed using real-time RT-PCR to detect chemokine expression. The relative expression levels of CCL2 and CCL20 chemokine mRNA in mouse skin increased by approximately 2-fold and 4-fold, respectively, six hours after 1270 nm laser irradiation. Treatment of human umbilical vein endothelial cells with near-infrared light irradiation at wavelengths of 1064 nm and 1270 nm, respectively, significantly promoted the synthesis of the endogenous bioactive molecule nitric oxide (NO), which has been shown to be an important member of the reactive oxygen species (ROS) family, in the cells (121). NO exerts regulatory effects on mitochondrial bioenergetics through multiple mechanisms. Experimental evidence indicates that NO interferes with electron transport chain activity, particularly by suppressing electron flux within the mitochondrial respiratory complexes. This inhibition paradoxically correlates with elevated oxygen utilization rates, possibly through a mechanism involving increased mitochondrial membrane polarization (122). Furthermore, nitric oxide exhibits vasodilatory effects on the lymphatic system via specific signaling pathways (123) and may significantly contribute to the structural expansion of lymphatic microvessels situated within the dermal tissue layer.

In summary, NIR at 1270 nm wavelength effectively stimulates mitochondrial biosynthesis of reactive oxygen species and adenosine triphosphate, which enhanced the vaccine adjuvant effect through light reception. This wavelength showed moderate to long-lasting antibody titers making it a promising candidate for clinical applications due to its low energy requirement and unique adjuvant effect (see **Table 1**).

**Table 1:** Summary of cupping, acupuncture, and near infrared light experiments.

Samples	Methods	Results and Conclusion	Reference
GFP plasmid, SARS-COV-2 DNA vaccine, prague-Dawley male rats (NTAC-SD; murine pathogen free), aged 7 to 10 weeks	In order to optimize the injection process through vacuum-assisted methodology, three aspiration mechanisms featuring distinct configurations were employed to establish a negative pressure environment. The operational parameters, including pressure intensity and duration, were precisely regulated in accordance with the predetermined experimental protocols to ensure process compatibility with varying research requirements.	Upon analysis of the experimental data, the geometric mean titer (GMT) level of humoral immune response after application of the suction device ranged between 2×10^3 and 5×10^3, presenting a statistically significant enhancement compared to the injection-only group (p<0.05).Notably, the immune response characteristics of rodents in the single injection combined with suction manipulation group showed a high degree of similarity with those of experimental animals in the secondary injection group. Histological assessment by hematoxylin-eosin (HE) staining method showed that none of the samples from the experimental group showed local histopathological damage or abnormal lymphocyte aggregation.	(62)
Wistar rats, SPF Females, weighing 230g–280g (10 weeks old)	A total of 40 female Wistar rats were randomly assigned to four experimental cohorts with equivalent numbers (n=10 per group). The intervention protocols were designed as follows: Cohort A received mock immunization combined with acupuncture stimulation, Cohort B underwent mock immunization with non-acupuncture intervention, Cohort C was subjected to active immunization accompanied by acupuncture treatment, while Cohort D served as the active immunization control group without acupuncture. Throughout the 6-week experimental period, animals in Groups A and B received daily subcutaneous administration of d-galactose (350 mg/kg/day), whereas counterparts in Groups C and D were administered equivalent volumes of physiological saline through the same route. This experimental design maintained consistent injection volumes and frequency across all study groups.	Following acupuncture intervention, marked elevations were observed in diphtheria antibody concentrations, lymphocyte subset proportions (specifically CD4+/CD8+ T-cell indices), and heat shock protein 70 gene transcriptional activity. These findings indicate the potential application of acupuncture-based interventions as innovative immunological enhancers in vaccination strategies. Notably, the experimental design incorporated a commercial DTaP preparation containing aluminum-based adjuvants, thereby demonstrating the compatibility of acupuncture therapy with conventional adjuvant systems. The investigation did face methodological constraints, including the absence of quantitative analysis for HSP70 protein synthesis and insufficient temporal resolution to delineate phase-specific immunological activation patterns throughout the immunization process.	(127)
161 female 8-week-old C57BL/6J mice (Japan SLC, Shizuoka, Japan)	Employing the murine P-815 mastocytoma cell line model, intracellular reactive oxygen species generation was quantified using the H2DCFDA fluorescent probe. Cellular fluorescence intensity was systematically assessed following laser exposure through flow cytometric analysis. Furthermore, adenosine triphosphate concentrations in both intact P-815 cells and mitochondrial fractions were precisely measured through the luciferin-luciferase enzymatic assay system. This dual experimental approach enabled comprehensive evaluation of oxidative stress dynamics and bioenergetic status under photostimulation conditions.	The 1270 nm wavelength within the near-infrared spectrum demonstrates immunostimulatory properties through photonic reception mechanisms. Experimental studies reveal that mitochondrial activation under this specific radiation involves concurrent generation of ROS and ATP biosynthesis. This dual metabolic response correlates with enhanced immunopotentiation when employed as a vaccine adjuvant. Notably, longitudinal analyses of humoral immunity parameters demonstrate that photostimulation at this wavelength induces sustained serological responses characterized by stable-to-elevated immunoglobulin concentrations over extended observation periods.	(128)

### 3.3 Diet and lifestyle interventions

A proper diet and lifestyle can significantly enhance the activation and responsiveness of the immune system, establishing a robust foundation for immune health (124, 125).That high-fat diets and circadian shifts can lead to reduced levels of B-lymphocytes and impaired immune function, adversely affecting the immune system. A high-fat diet significantly affects intestinal immune function and may induce impaired intestinal barrier function, which is a potential causative factor for intestinal inflammation and inflammation-related diseases. A low-carb, high-protein diet reduces weight growth rates and lipid levels, regulates immune indicators, and improves the structure of the gut microbiota.

Ensuring adequate intake of essential vitamins (e.g. vitamins C, D) (126) and minerals (e.g. zinc, iron) helps support the immune system. For example, as an essential micronutrient, ascorbic acid (vitamin C) plays an important role in enhancing the body’s immune response and inhibiting pathogen attack through its remarkable antioxidant properties. High-fiber foods, such as whole grains, vegetables, and fruits support gut health, which indirectly enhances the immune response. Engaging in regular physical activity at appropriate intensity levels has been shown to enhance immune system functionality by promoting the proliferation of anti-inflammatory cellular components within the organism. Conversely, inadequate sleep duration or suboptimal sleep quality may compromise immunological defenses, resulting in heightened susceptibility to pathogenic agents and infectious processes. Chronic stress leads to elevated cortisol levels, which can suppress the immune system. Adopting effective stress management strategies such as meditation and yoga can help reduce stress and boost immunity. See **Table 2**, in order to support immune activation and overall health, proper eating habits and a healthy lifestyle play a crucial role.

**Table 2:** Benefits of dietary and lifestyle approaches to the immune system.

Methods	Benefits
Consumption of antioxidant-rich foods such as citrus fruits, berries, nuts and green leafy vegetables	Helps reduce oxidative stress and protects immune cells from free radical damage
Increase intake of prebiotics and probiotics, e.g. through consumption of yoghurt, fermented foods and probiotic supplements	Maintains the balance of the intestinal microbiota, strengthens the intestinal barrier function, and promotes the development of the immune system
High-quality protein sources such as lean meat, fish, pulses and eggs	Provides essential amino acids to support antibody production and immune function
Practice meditation, yoga or other relaxation techniques	Helps relieve stress and strengthens the immune system
Exercise regularly and don’t overtrain	Improving the efficiency of the immune system

## 4 Impact of environmental exposure and immune memory on vaccine response

### 4.1 The role of immune memory regulation

Immune memory induced by previous infections or vaccinations can significantly enhance the response to vaccines against homologous pathogens. For example, booster shots of COVID-19 vaccine stimulate rapid proliferation of memory B cells, leading to a substantial increase in antibody titers (129, 130). In particular, heterologous booster immunization strategies (e.g., mRNA vaccine followed by RBD-HR/trimer vaccine) show stronger cellular immune responses and longer-lasting memory responses than homologous boosts (130). The phenomenon of immune imprinting allows the immune system to generate a faster and stronger response to a previously exposed pathogen or vaccine (131). This memory effect is also seen in influenza vaccines, where previous infections can enhance the antibody response to inactivated influenza vaccines and help achieve protective antibody levels (132).

Original Antigenic Sin (OAS) may result in a limited response to a heterologous vaccine. For example, if there is a significant mutation in the pathogen (e.g., influenza virus or SARS-CoV-2 variant), the immune system may preferentially respond against the original strain rather than the new variant, thereby decreasing protection against the new variant (131, 133). In some cases, prior infection may result in a non-optimal vaccine response. For example, the dengue vaccine may pose a risk of antibody-dependent enhancement (ADE) in previously infected individuals (133).

### 4.2 Impact of living conditions and geographical climate

Populations in low pathogen exposure settings (e.g., developed countries) may exhibit a Th2 response bias, increasing the risk of sensitization and weakening anti-infection immunity (134). Conversely, populations in high pathogen exposure environments (e.g., the tropics) may exhibit stronger Th1/IL-10 ratios, which may enhance vaccine efficacy but may also exacerbate inflammatory responses (134). UV radiation may inhibit the function of cutaneous Langerhans cells, thereby reducing the efficiency of transdermal vaccines (135). For example, HPV vaccines may exhibit lower antibody positivity rates in areas with higher UV exposure.

## 5 Summary and outlook

This review examines how a variety of factors influence vaccine immunogenicity, focusing on the impact of host-intrinsic factors, external intervention strategies, and external environmental factors on vaccine efficacy. The findings highlight the importance of these factors in vaccine response and provide a basis for further optimization of vaccine design and personalized vaccination strategies.

Although existing studies provide a rich theoretical foundation and practical basis for personalized vaccine strategies in terms of host-intrinsic factors, external intervention strategies, and the external environment, the integration of these multidimensional factors still faces many challenges. Firstly, synergistic or antagonistic effects between different therapies have not been clarified. For example, acupuncture and moxibustion regulate immune homeostasis through anti-inflammatory mechanisms, whereas the effectiveness of vaccines often relies on moderate inflammatory activation to initiate an immune response. This paradox requires us to more finely regulate the timing and intensity of the application of adjuvant therapies to ensure that their immune-boosting effects are achieved without diminishing the effectiveness of the vaccine. Specifically, for acupuncture, adjuvants should be used strategically after the initial proinflammatory phase of the vaccine response (usually 24–48 hours postvaccination) to allow the vaccine to first trigger the necessary innate immune activation (136). Dose-control regimens (e.g., shorter needle retention time, lower moxibustion intensity) can further balance anti-inflammatory modulation while maintaining vaccine efficacy (137). More importantly, although adjuvant therapies such as acupuncture, moxibustion, and suction-based skin delivery (derived from cupping principles) have shown potential to modulate immunity and enhance vaccine response in preclinical models and small clinical observations, the exact validation of their core mechanisms of action in humans still faces significant challenges. A key limitation is the paucity of rigorously designed large-scale randomized controlled trials (RCTs), which are essential to conclusively demonstrate the specific mechanisms, strength of effect, and safety of these therapies in optimizing the effectiveness of vaccination in populations.

Secondly, most of the existing studies focus on the effect of a single factor and lack a systematic exploration of the interaction of multiple factors. Questions such as how the interaction of gut flora and genetic metabolism affects vaccine response, or whether the combination of adjuvant therapies and novel delivery techniques can have a stacking effect, have not been adequately answered. Future research should leverage multi-omics technologies (e.g., single-cell RNA sequencing to resolve immune cell heterogeneity) and artificial intelligence (e.g., machine-learning algorithms) to integrate genetic, metabolic, and immune multidimensional data to construct predictive models to guide personalized regimens (e.g., predicting an individual’s antibody response to a specific vaccine or optimal adjuvant selection).

The development of personalized immuno-optimized vaccines is a rapidly growing area in terms of future research directions. With a deeper understanding of genetic metabolism, delivery methods and adjuvant therapies, based on the existing research progress, gene editing technology represented by CRISPR-Cas9 shows potential application value in the field of vaccine development. It is expected to optimize the efficacy of vaccines and significantly reduce the incidence of adverse reactions by precisely regulating individual-specific immune response mechanisms. CRISPR-Cas9 can assist in optimizing the sequence design of mRNA vaccines. CRISPR screening is used to identify key sequences (e.g., UTR regions) that affect mRNA stability and translation efficiency, and then to design mRNA vaccines with high expression of antigens (138). For example, the mRNA vaccine against SARS-CoV-2 spiking protein reduced the GC content by editing the secondary structure of the 5’-UTR, which led to an increase in protein expression and a significant enhancement of neutralizing antibody titer (138). Using AI and big data to analyze patient-specific tumor mutations or antigenic profiles of infectious agents to design more personalized vaccines. Modern science and technology, such as biosensors and imaging techniques, are used to monitor changes in physiological parameters during cupping, acupuncture and moxibustion treatments. NIR phototherapy is expected to promote immune cell activity by improving the supply of oxygen to local tissues.
